# A Case of Milroy's Disease

**Published:** 1912-02-10

**Authors:** J. Bernard Dawson


### A CASE OF MILROY'S DISEASE.
### With Notes on Trophœdema.

By J. BERNARD DAWSON, M.D. Lond., F.R.C.S.

When confronted with a case of œdema the medical man's first thought is to discover the cause. In the great majority of cases this is not difficult, for clinical investigation soon decides whether hæmic, cardiac, pulmonary, renal, or hepatic mischief is at the bottom of the pathological condition. There are, however, cases in which no *fons et origo* can be found, and the physician is compelled to seek for some other explanation. Of late years there have appeared in medical literature accounts of patients subject to either transitory or persistent œdema of various parts of the body whose physical examination gives no clue to the underlying cause. These have been considered to be due to some derangement of the vasomotor mechanism, and have been called the trophœdemata. How far this hypothesis is correct is uncertain, so that the writer whilst adopting this nomenclature does so with the reservation that future investigation may prove it unsuitable.

### The Patient.

It is the object of this communication to relate the notes of a case of persistent hereditary œdema, or Milroy's disease, and then to show to what extent it is correlated to the other forms of neurotic œdemata.

The patient is a healthy girl of eighteen, unmarried, and living an outdoor life in the country. She is well nourished, and has had no illness save an infantile attack of measles. She commenced to menstruate at fifteen, and has done so regularly since. She is regular in her daily hygiene, and does not suffer from constipation. Her mother, two sisters, and a brother are living and healthy; her father is mentioned fully below.

Five years ago the patient's ankles were noticed to be swollen, and apparently neither started prior to the other. This swelling has progressed slowly, steadily, and painlessly, until the present date. Her legs are now swollen as far as the knees, where the œdema stops abruptly. The swelling is of the type of solid œdema, smooth, cool, firm, without visible engorgement of blood or lymphatic vessels, and symmetrical. The swollen legs pit on firm pressure, but much greater force is needed than in the ordinary cardiac or renal form. There is no evidence of venous obstruction either in the groin, the pelvis, or the abdomen. The lymphatics are apparently unaffected by any gross lesion.

### Further Symptoms.

The pulse is 72 per minute, regular in volume and rhythm, of moderate tension, and in a normal vessel. The heart is normal in size and action, whilst the blood is as follows: Hæmoglobin 80 per cent., red corpuscles 4,450,000 per cmm., white corpuscles 7,000, normal in quality and relative quantity. The examination of a blood film reveals absolutely no abnormality of any corpuscle. The urine is acid, of specific gravity 1015, containing neither albumen nor sugar; there is no deposit, and microscopic examination was fruitless.

Although the girl is in no way physically inconvenienced, she suffers from periods of mental depression, and is very sensitive about her trouble, so much so that she unfortunately refuses to be photographed. The entire absence of any cause for the œdematous condition suggested the diagnosis of persistent hereditary œdema, or Milroy's disease, in spite of the absence of any other case in her family.

### Family History.

The possibility of this being a sporadic case then suggested itself, but a further careful inquiry into the family history was instituted. The child's mother then stated that her husband had died some years before in an asylum, and that he had been "swollen." This evidence, though slender, was helpful, for the disease is associated with mental instability. Nothing more could be ascertained as to the patient's direct or collateral ancestors.

Milroy, of Omaha, first called attention to the disease in a paper entitled "An undescribed variety of Hereditary Œdema," published in 1892 in the *New York Medical Journal*, since when other observers, notably Meige and Rolleston, have written upon the subject. In Milroy's original case there were in one family twenty-two cases in six generations, whilst in the *Quarterly Journal of Medicine* for April 1908 Hope and French published a case with a history of thirteen affected members in five generations of a family.

The above description gives a fair idea of the condition in a fairly early stage, but there are several other phenomena which have been manifested by other patients. The œdematous condition tends gradually to extend, but always to show an abrupt demarcation at a joint; thus in the earliest the ankle is the upper limit, later the knee, and still later the groin at Poupart's ligament. The swelling seems to be temporarily arrested at these points and to spread steadily between them. The arms and the trunk of the body are not affected, but Osler has described a case of vasomotor neurosis characterised by swelling and tumefaction of the whole arm on exertion.

### Acute Attacks.

The most striking phenomena described are the acute attacks; in Hope and French's case there were in all ten exacerbations or pain and increased swelling, the chief features of which were sudden onset with rigor; elevation of temperature, rapidity of pulse and respiration; pain and swelling in the pudenda; pain and increased swelling of the legs; short duration of from five to seven days; occurrence on affected legs of superficial ulcers; and definite constitutional distress, notably vomiting. In fact these attacks bear a marked resemblance to an outbreak of angio-neurotic œdema, a point which will be referred to more fully later.

### The General View.

The entire absence of any lesions that might account for the phenomena of Milroy's disease has forced writers on the subject to adopt the view that they are due to a change in the vasomotor machinery regulating the vessels of the legs. This view must be regarded as an "asylum ignorantiæ," for it is merely an assumption that all is not well with a piece of nervous apparatus of the functions of which we have very scanty knowledge. It is, however, supposed that the nervous control of the vessels is deficient, and that this allows an undue and unusual amount of transudation into the tissues. Whatever be the true cause, it seems obvious that it is local in effect, for the œdema is strictly local in its occurrence.

The three chief causes which might produce such a condition are:—

(*a*) Venous obstruction, due to pressure, thrombosis, etc.
(*b*) Lymphatic obstruction.
(*c*) Vasomotor disturbance.

With regard to the first suggestion, there is some interest aroused by the occurrence of the acute attacks, which undoubtedly resemble a microbic infection which might implicate the veins, producing a thrombosis. Against this theory there are arrayed formidable facts—viz., the absence of any venous engorgement, the undisturbed temperature of the leg, the maintenance of a normal circulation in the limb, and the abrupt limitation of the swelling.

### Lymphatic Obstruction.

The hypothesis of lymphatic obstruction seems to be negatived by the fact that the swelling always appears before the onset of the acute attacks, and by the fact that these attacks are not present in every case.

In order to adopt with some confidence the last, or nervous, view, it is helpful to compare the phenomena of Milroy's disease with those of other diseases usually attributed to vasomotor influences. These are:—

(*a*) Raynaud's disease.
(*b*) Erythromelalgia.
(*c*) Factitious urticaria.
(*d*) Angio-neurotic œdema.

The last two named conditions are probably the same disease showing some variation in degree.

All these affections have certain points of similarity both with themselves and with Milroy's disease, chief among which is the hereditary element. Each of them is either directly to be traced in successive generations, or they are to be found in the descendants of people who have been subject to manifestations of nervous instability; this feature is very marked in the case of Milroy's disease, a point of importance in attempting to bring this disease into line with the vasomotor neuroses.

### A Resemblance.

The acute attacks which occurred in Hope and French's case of Milroy bear a striking resemblance to the onset of angio-neurotic œdema. Thus in both diseases there occur sudden œdema associated with pain, sometimes with temperature, and with mild gastro-intestinal symptoms. The abrupt and rapid subsidence of the exacerbation is another point of resemblance. Similarly the superficial ulceration which has occurred during an acute Milroy attack suggests the localised gangrene of Raynaud's disease. The phenomena attending an attack of the rare disease of erythromelalgia bear a striking resemblance to the acute Milroy attacks, the only marked difference being the occurrence of sensory symptoms in the former. A combination of the symptoms of erythromelalgia and angio-neurotic œdema gives a clinical picture very closely resembling that of an acute Milroy attack—viz., local heat and pain, constitutional disturbance indicated by elevation of temperature, etc., gastro-intestinal phenomena, hereditary tendency, recurrence and rapid decline of symptoms.

The association of this group of diseases with definite nervous disorders is also suggestive, for we find that lesions resembling Raynaud's disease occur in peripheral neuritis and syringo-myelia, and erythromelalgia is frequently associated with disseminated sclerosis, tabes, myelitis, and syringo-myelia.

### Previous Nervous Trouble.

Lastly, in Milroy's disease there is often some history of nervous trouble in the patient or her relatives. Thus in the case recorded above the girl's father was insane, whilst in Hope and French's series there were six examples of mental derangement, including epilepsy and dipsomania.

Reviewing this group of diseases as a whole, it is obvious that there is some connection between its components, for in all there occurs a causeless œdema, persistent or transitory, with which are associated certain pathological phenomena which vary in their degree and incidence in the individual.

Reverting to the case of Milroy's disease quoted above, it only remains to say a word as to treatment.

### A Final Word on Treatment.

This is very unsatisfactory, and consists of careful and continuous bandaging. Medicine is useless, although obvious constitutional debility should be attended to. The importance of the bandages cannot be too much emphasised, for neglect of this permits of swelling, which incapacitates the patient.

There are several instances of men who have been able to enjoy a long life of hard work without serious discomfort, so long as their legs were well supported. The œdema is slightly reduced by rest in bed, and it would probably be desirable to subject a patient to a week's recumbency before applying the bandages.

